# Uptake of Community-Based Peer Administered HIV Point-of-Care Testing: Findings from the PROUD Study

**DOI:** 10.1371/journal.pone.0166942

**Published:** 2016-12-02

**Authors:** Lisa Lazarus, Sheetal Patel, Ashley Shaw, Sean Leblanc, Christine Lalonde, Manisha Hladio, Kira Mandryk, Cynthia Horvath, William Petrcich, Claire Kendall, Mark W. Tyndall, for the Proud Community Advisory Committee

**Affiliations:** 1 Ottawa Hospital Research Institute, Ottawa, Canada; 2 PROUD Community Advisory Committee, Ottawa, Canada; 3 Drug Users Advocacy League, Ottawa, Canada; 4 Department of Medicine, University of Ottawa, Ottawa, Canada; 5 Ottawa Public Health, Ottawa, Canada; 6 CT Lamont Primary Health Care Research Centre, Bruyere Research Institute, Department of Family Medicine, University of Ottawa, Ottawa, Canada; 7 University of Ottawa at the Ottawa Hospital, Division of Infectious Diseases, Ottawa, Canada; University of New South Wales, AUSTRALIA

## Abstract

**Objectives:**

HIV prevalence among people who inject drugs (PWID) in Ottawa is estimated at about 10%. The successful integration of peers into outreach efforts and wider access to HIV point-of-care testing (POCT) create opportunities to explore the role of peers in providing HIV testing. The PROUD study, in partnership with Ottawa Public Health (OPH), sought to develop a model for community-based peer-administered HIV POCT.

**Methods:**

PROUD draws on community-based participatory research methods to better understand the HIV risk environment of people who use drugs in Ottawa. From March-October 2013, 593 people who reported injecting drugs or smoking crack cocaine were enrolled through street-based recruitment. Trained peer or medical student researchers administered a quantitative survey and offered an HIV POCT (bioLytical INSTI test) to participants who did not self-report as HIV positive.

**Results:**

550 (92.7%) of the 593 participants were offered a POCT, of which 458 (83.3%) consented to testing. Of those participants, 74 (16.2%) had never been tested for HIV. There was no difference in uptake between testing offered by a peer versus a non-peer interviewer (OR = 1.05; 95% CI = 0.67–1.66). Despite testing those at high risk for HIV, only one new reactive test was identified.

**Conclusion:**

The findings from PROUD demonstrate high uptake of community-based HIV POCT. Peers were able to successfully provide HIV POCT and reach participants who had not previously been tested for HIV. Community-based and peer testing models provide important insights on ways to scale-up HIV prevention and testing among people who use drugs.

## Introduction

Despite major gains in HIV prevention, treatment and care, uptake among people who use drugs (PWUD) remains inequitable [1]. HIV testing among at-risk groups is central to HIV prevention efforts, in particular as the ‘*treatment as prevention*’ paradigm has been shown to improve health outcomes and longevity among those infected, to prevent onward HIV transmission, and to reduce healthcare costs [2–5]. However, PWUD are more likely to receive HIV testing later in the course of infection and some only after developing HIV-related opportunistic infections [6]. This disparity to accessing conventional HIV testing services among PWUD reflects the structural, economic, social, and individual-level barriers experienced by this population [6], including perceived and enacted stigma by health care providers [7].

The recent availability of rapid HIV point-of-care testing (POCT) offers new strategies for improving the uptake of testing among PWUD in both clinical and community-based settings [8,9]. Rapid HIV POCT can reduce anxiety associated with waiting for test results: non-reactive results can be provided at the time of testing, while participants with reactive results can receive on-the-spot linkages to confirmatory testing, support, and treatment [10]. Community-based POCT offers the additional advantage of reaching high risk populations who might not otherwise seek care [11], especially if peers are incorporated into service delivery [6]. Training peer leaders who use (and continue to use) drugs as HIV educators has been shown to be an effective mechanism for HIV prevention among PWUD [12–16]. Furthermore, peer-delivery of testing has been delivered within communities of men who have sex with men (MSM) [11,17,18] and among PWUD with high levels of satisfaction and comfort with confidentiality [19].

The city of Ottawa, Ontario has a large network of people who use both injection and non-injection drugs for a city of its size [20–22]. Despite declines in HIV incidence among the general population, HIV rates among people who use injection drugs in Ottawa remain disproportionately high, with an estimated prevalence of 10% [21]. As 43% of PWUD who were diagnosed with HIV report sharing drug use equipment [21], ongoing transmission remains a significant concern. The PROUD Study is a community-based research project that arose in response to the high rates of HIV among PWUD in Ottawa. In this study, we report a novel community-based peer-administered POCT program developed in collaboration with the local public health authority and communities of PWUD. To the best of our knowledge, and with the exception of the integration of peer educators and testers in a pilot project in Vancouver’s Downtown Eastside [19], there are no other examples of engaging PWUD as testers in community-based HIV testing. Our objective was to elicit the factors associated with the uptake of community-based HIV POCT and to inform models of peer-administered testing among this population.

## Methods

### Study Design

The Participatory Research in Ottawa: Understanding Drugs (PROUD) Study is a prospective cohort study examining HIV risk among people who use drugs in Ottawa, Ontario and has been described elsewhere [23]. Briefly, between March and December 2013, eligible and consenting participants completed a one-time interviewer-administered iPad-based questionnaire, followed by an opt-in HIV POCT. The quantitative survey included individual-level variables, including socio-demographic information and drug use patterns; interpersonal variables, including sexual history and connections to community; and structural variables, including access and use of harm reduction services, information on housing and homelessness, experiences with the law, and health status and access to health care, including hepatitis C virus (HCV) and HIV testing and treatment. Interviews were administered by a peer or medical student/community ally, depending on interviewer availability and participant comfort. All interviewers received comprehensive training in research ethics, including confidentiality and privacy, and interviewing skills. A cash honorarium (CAN $20.00) was offered after survey completion regardless of whether participants chose to opt-in to HIV POCT.

### Study Participants

As previously described in detail [23], potential participants were recruited through peer-based purposive recruitment on the streets and in social service settings frequented by PWUD. Interviews were conducted at our research site, located in Ottawa’s downtown Byward Market area. The space, rented for the purpose of this study, used to be a drop-in centre and was therefore well-known to the community and centrally located. Eligibility criteria included age 16 years or older, having injected or smoked drugs other than marijuana in the past 12 months, and having lived in Ottawa for at least three months.

### POCT Intervention

Interviewers (eleven peers, fifteen medical students, and three community allies) received HIV POCT (bioLytical INSTI test) training and certification by Ottawa Public Health. Training included sessions on the following topics: therapeutic relationships and confidentiality, HIV 101, infection control and quality assurance, performing the POCT and result interpretation, and pre- and post-test counseling (including role playing and debriefing). A public health nurse with harm reduction experience was onsite to assist with pre- and post-test counseling, conducting the POCT as well as drawing confirmatory serology for reactive or indeterminate POCT results, and to provide quality assurance, support, referrals and debriefing with peers and medical students. POCT was offered by the survey interviewer upon survey completion. Participants were excluded from POCT if the public health nurse was not present, if they responded ‘yes’ to the question ‘Have you ever tested positive for HIV?’ during the interview-administered questionnaire, or if they declined for other reasons (with reason recorded by interviewers).

This study received ethical approval from The Ottawa Hospital and Ottawa Public Health Research Ethics Boards. All participants provided their written consent to participate in the study and the consent process was approved by both REBs.

### Statistical Analysis

We undertook descriptive and univariate analyses [24] to examine which patient characteristics were associated with agreement to undergo HIV POCT, including: drug use patterns; injection practices; equipment sharing practices; access to health care; past experiences with HIV and HCV testing; condom use with sexual partners; sex work; housing status; and experiences with the law. The exposures of interest consisted of those study questions that the investigators and/or peers determined to be potentially associated with perceived need for testing and barriers to accessing care for PWUD. We used chi-square or Fisher exact tests as appropriate for categorical variables and the Wilcoxon rank sum test for continuous variables. Associations are presented as univariate odds ratios with corresponding 95% confidence intervals (CIs).

## Results

593 participants were recruited for this analysis: 5 (0.8%) were excluded due to the unavailability of POCT and onsite nursing on the day of their interview and 38 (6.4%) were excluded because of self-reported HIV infection. Of those remaining, 550 (92.7%) were offered HIV POCT: Peers and medical student/allies offered testing to 233 (42.4%) and 317 (57.6%) participants respectively. There was no significant difference in uptake of testing between those offered a peer vs. a non-peer interviewer (83.7% vs. 83.0% respectively, OR = 1.05; 95% CI = 0.67–1.66). The most common reasons for testing refusal were no specific reason provided (n = 21, 22.8%) and recent HIV testing (n = 20, 21.7%). Among participants who consented to POCT, 74 (16.2%) reported never having previous HIV testing.

Table 1 shows the demographic and drug use characteristics of the participants by uptake of POCT. Participants who agreed to POCT were older (43 vs. 39 years, p = 0.02) and less likely to be female (OR = 0.59; 95% CI = 0.35–0.98). Participants who reported ever injecting drugs were more likely to have consented to testing (OR = 1.71 95% CI = 1.09–2.69), but there were no other significant differences in drug use characteristics between the two groups.

**Table 1:** Demographic and drug use characteristics associated with uptake of an HIV POCT^a^.

Characteristic	Uptake of an HIV POCT No. (%) of participants^b^		OR (95% CI)
	Yes n = 458	No n = 92	
**Age**^c^ **(median, IQR)**	43 (34–50)	39 (30–48)	**P-value = 0.02**
**Gender**			
Female	87 (77.0)	26 (23.0)	**0.59 (0.35–0.98)**
Male	366 (85.1)	64 (14.9)	*ref*
**Language**			
French	80 (81.6)	18 (18.4)	0.81 (0.45–1.44)
English	341 (84.6)	62 (15.4)	*ref*
**Education**			
Less than high school	228 (83.5)	45 (16.5)	1.04 (0.66–1.62)
High school and higher	230 (83.0)	47 (17.0)	*ref*
**Ethnicity**			
Aboriginal	84 (82.3)	18 (17.7)	0.92 (0.52–1.63)
Non-Aboriginal	374 (83.5)	74 (16.5)	*ref*
**Neighbourhood of Residence**			
Market	197 (81.1)	46 (18.9)	0.73 (0.46–1.16)
Other Ottawa neighbourhood	240 (85.4)	41 (14.6)	*ref*
**Sexual Identity**			
LGBQ^d^	32 (74.4)	11 (25.6)	0.53 (0.25–1.09)
Heterosexual	421 (84.7)	76 (15.3)	*ref*
**Ever Injected Drugs**			
Yes	300 (86.0)	49 (14.0)	**1.71 (1.09–2.69)**
No	154 (78.2)	43 (21.8)	*ref*
**Injected Drugs in the past 12 months**^e^			
Yes	198 (84.6)	36 (15.4)	0.65 (0.33–1.31)
No	101 (89.4)	12 (10.6)	*ref*
**Injected Cocaine**			
Yes	129 (84.3)	24 (15.7)	0.95 (0.45–2.01)
No	68 (85.0)	12 (15.0)	*ref*
**Injected Crack cocaine**			
Yes	99 (86.8)	15 (13.2)	1.38 (0.67–2.84)
No	96 (82.8)	20 (17.2)	*ref*
**Injected Opiates**			
Yes	156 (83.9)	30 (16.1)	0.74 (0.29–1.90)
No	42 (87.5)	6 (12.5)	*ref*
**Injected Speedball**			
Yes	53 (85.5)	9 (14.5)	1.11 (0.49–2.52)
No	143 (84.1)	27 (15.9)	*ref*
**Injected with other people**			
Yes	164 (85.9)	27 (14.1)	1.76 (0.76–4.11)
No	31 (77.5)	9 (22.5)	*ref*
**Injected in public**			
Yes	65 (78.3)	18 (21.7)	0.50 (0.24–1.04)
No	122 (87.8)	17 (12.2)	*ref*
**Assistance to inject**			
Yes	73 (82.0)	16 (18.0)	0.74 (0.36–1.52)
No	123 (86.0)	20 (14.0)	*ref*
**Used unknown needle**			
Yes	32 (88.9)	≤ 5 (11.1)	1.56 (0.52–4.72)
No	164 (83.7)	32 (16.3)	*ref*
**Injected with used needle**			
Yes	43 (84.3)	8 (15.7)	0.98 (0.42–2.31)
No	153 (84.5)	28 (15.5)	*ref*
**Ever Overdosed**			
Yes	193 (83.6)	38 (16.4)	1.03 (0.65–1.63)
No	261 (83.1)	53 (16.9)	*ref*

OR = odds ratio, CI = confidence interval.

^a.^ Percentages are calculated on the basis of the sum across each row.

^b.^ Except where indicated otherwise. Due to ‘no answer’/missing responses, the data for some characteristics do not sum to n = 550.

^c.^ Median age based on n = 522.

^d.^ LGBQ stand for Lesbian, Gay, Bisexual and Queer.

^e.^ All drug use behaviour variables are reported for the previous 12 months unless otherwise specified.

Table 2 presents health status and access to health and harm reduction services among those who agreed and did not agree to HIV POCT. No significant differences were found between the two groups.

**Table 2:** Health characteristics and access to health and harm reduction services associated with uptake of an HIV POCT^a^.

Characteristic	Uptake of an HIV POCT No. (%) of participants^b^		OR (95% CI)
	Yes n = 458	No n = 92	
**Ever trouble accessing new needles**			
Yes	33 (84.6)	6 (15.4)	0.96 (0.37–2.50)
No	171 (85.1)	30 (14.9)	*ref*
**Accessed addiction treatment past 12 months**			
Yes	174 (86.1)	28 (13.9)	1.36 (0.84–2.22)
No	278 (82.0)	61 (18.0)	*ref*
**Ever received support from a peer worker**			
Yes	182 (85.4)	31 (14.6)	1.29 (0.80–2.07)
No	273 (82.0)	60 (18.0)	*ref*
**Ever received support from other organizations**			
Yes	273 (84.0)	52 (16.0)	1.10 (0.69–1.73)
No	182 (82.7)	38 (17.3)	*ref*
**Attempted suicide past 12 months**			
Yes	39 (95.1)	≤ 5 (4.9)	4.00 (0.95–16.87)
No	410 (83.0)	84 (17.0)	*ref*
**Sought care for health issue in past 12 months**			
Yes	270 (85.4)	46 (14.6)	1.42 (0.90–2.23)
No	186 (80.5)	45 (19.5)	*ref*
**Sought care from a hospital or ER in past 12 months**			
Yes	183 (86.7)	28 (13.3)	1.35 (0.71–2.58)
No	87 (82.9)	18 (17.1)	*ref*
**Have regular doctor**			
Yes	243 (85.6)	41 (14.4)	1.38 (0.88–2.18)
No	214 (81.1)	50 (18.9)	*ref*
**Ever tested for HCV**			
Yes	378 (84.4)	70 (15.6)	1.18 (0.63–2.22)
No	64 (82.0)	14(18.0)	*ref*
**Result of last HCV test**			
Positive	160 (86.0)	26 (14.0)	1.28 (0.75–2.18)
Negative	193 (82.8)	40 (17.2)	*ref*
**Know that can be charged for not disclosing HIV status to partners**			
Yes	397 (83.4)	79 (16.6)	1.04 (0.53–2.03)
Other (No, Maybe, Don’t know)	58 (82.9)	12 (17.1)	*ref*
**Ever tested for HIV**			
Yes	369 (82.2)	80 (17.8)	0.62 (0.31–1.26)
No	74 (88.1)	10 (11.9)	*ref*
**Tested for HIV in the past 12 months**			
Yes	187 (82.7)	39 (17.3)	1.06 (0.63–1.77)
No	145 (81.9)	32 (18.1)	*ref*
**Worried about HIV exposure**			
Yes	191 (85.7)	32 (14.3)	1.37 (0.85–2.21)
No	239 (81.3)	55 (18.7)	*ref*
**Who administered HIV test**			
Peer	195(83.7)	38 (16.3)	1.05 (0.67–1.66)
Other (medical student, nurse, RA)	263 (83.0)	54 (17.0)	*ref*

OR = odds ratio, CI = confidence interval

^a.^ Percentages are calculated on the basis of the sum across each row

^b.^ Except when indicated otherwise. Due to ‘no answer’/missing responses, the data for some characteristics do not sum to n = 550.

Table 3 shows variables related to structural risks including associations with the law, sex work, and housing status. Participants who reported having engaged in sex work in the past 12 months were significantly less likely to agree to HIV POCT (OR = 0.48; 95% CI = 0.25–0.92), whereas participants who reported having spent overnight or longer in jail in the past 12 months or to have consumed drugs in jail were significantly more likely to agree to testing (OR = 1.71; 95% CI = 1.05 to 2.78) and (OR = 2.43; 95% CI = 1.07–5.52), respectively.

**Table 3:** Legal system encounters, sex work, and housing characteristics associated with uptake of an HIV POCT^a^.

Characteristic	Uptake of an HIV POCT No. (%) of participants^b^		OR (95% CI)
	Yes n = 458	No n = 92	
**Sex partner in past 12 months**			
Yes	338 (84.9)	60 (15.1)	1.37 (0.84–2.25)
No	115 (80.4)	28 (19.6)	*ref*
**Condom use in past 12 months**			
Not always	223 (85.4)	38 (14.6)	1.12 (0.36–3.44)
Always	21 (84.0)	≤ 5 (16.0)	*ref*
**Gave drugs, $, gifts for sex**^c^			
Yes	63 (85.1)	11(14.9)	1.02 (0.41–2.52)
No	62 (84.9)	11 (15.1)	*ref*
**Engaged in sex work**			
Yes	44 (74.6)	15 (25.4)	**0.48 (0.25–0.92)**
No	343 (86.0)	56 (14.0)	*ref*
**Current place of residence**			
Unstably housed	277 (82.2)	60 (17.8)	0.80 (0.50–1.28)
Stably housed	179 (85.2)	31 (14.8)	*ref*
**Ever been homeless**			
Yes	405 (83.2)	82 (16.8)	0.85 (0.41–1.80)
No	52 (85.3)	9 (14.7)	*ref*
**Homeless for at least one night in past 12 months**			
Yes	345 (85.5)	68 (16.5)	1.08 (0.65–1.80)
No	113 (82.5)	24 (17.5)	*ref*
**Ever redzoned**^d^			
Yes	177 (85.1)	31 (14.9)	1.21 (0.75–1.95)
No	278 (82.5)	59 (17.5)	*ref*
**Stopped/searched by police**			
Yes	302 (84.1)	57 (15.88)	1.09 (0.68–1.76)
No	155 (82.9)	32 (17.1)	*ref*
**Kept overnight or longer in jail**			
Yes	202 (87.8)	28 (12.2)	**1.71 (1.05–2.78)**
No	253 (80.8)	60 (19.2)	*ref*
**Consumed drugs in jail**			
Yes	116 (92.1)	10 (7.9)	**2.43 (1.07–5.52)**
No	86 (82.7)	18 (17.3)	*ref*

OR = odds ratio, CI = confidence interval.

^a.^ Percentages are calculated on the basis of the sum across each row.

^b.^ Except where indicated otherwise. Because of ‘no answer’/missing responses, the data for some characteristics do not sum to n = 550.

^c.^ Variables are reported for the previous 12 months unless otherwise specified.

^d.^ Redzones were defined as any geographical area or neighborhood where law enforcement had restricted the participant’s movement.

Only one POCT resulted as reactive and was subsequently confirmed as HIV positive with confirmatory serology.

## Discussion

This study demonstrates the high uptake of a community-based HIV POCT model for PWUD. In order to optimally reach people who use drugs with HIV prevention efforts, our findings support the expansion of novel community-based methods for providing testing, including peer-administered approaches. To the best of our knowledge, and with the exception of the integration of peer educators and testers in a pilot project in Vancouver’s Downtown Eastside [19], there are no other examples of engaging PWUD as testers in community-based HIV testing.

We found no difference in uptake of testing when HIV POCT was offered by a peer versus a non-peer interviewer, supporting previous work demonstrating that people at risk may be comfortable with having POCT administered by peers in a community setting. As has been demonstrated among MSM communities [11], peer testers in our study were able to handle tests, deliver non-reactive results, and provide pre- and post-test counseling in a manner that resonated with participants. Onsite nursing supervision provided peers with support for all aspects of HIV POCT, including: prompting on the POCT process; completing documentation; and guidance to identify when nurse involvement was appropriate. Furthermore, we successfully reached participants who had not previously been tested for HIV, thus providing new opportunities for HIV prevention education and counseling. Previous research has demonstrated that peer-delivered messaging has the potential to increase the interpretation and uptake of HIV prevention information by PWUD, providing value above POCT alone [25,26].

While there were few differences in characteristics among those who agreed and did not agree to HIV POCT, a few key findings emerged. There was high uptake of our community-based testing model among PWUD at high risk of HIV infection, including those who use injection drugs [27] and those reporting drug use while incarcerated [28]. These findings corroborate those of a Baltimore study that found high opt-in rates for onsite rapid HIV testing at probation and parole offices [10]. In our study, testing followed an hour-long interview, and honoraria were provided regardless of whether or not testing occurred, thus testing rates likely reflect a strong desire to be tested among individuals who have previously been incarcerated. However, low agreement for testing among female participants and those involved in sex work suggest the need for more targeted approaches to reach populations who may face multiple sources of stigma and experience increased barriers to accessing HIV testing and treatment services [29]. More specialized approaches, including female only services [29,30], should be explored to better reach these groups.

There are several limitations to our study. First, we used purposive sampling, which allowed us to target recruitment towards those facing multiple and intersecting oppressions among the study participants. However, our findings may not be applicable to all populations of PWUD. Second, characteristics are based on self-report to highly sensitive questions, which may have contributed to social desirability bias and an underreporting of high-risk practices. Third, it is possible that those who declined testing did so not because of the absence or presence of characteristics found to be associated with uptake of testing, but because they were aware of their positive status or had very recent testing, which was not fully elicited by our study. It is also possible that characteristics of testers other than peer or student status influenced uptake. For example, peers tended to be older and male, whereas medical students were younger and female. Participants were provided an option for who would conduct the test when possible, but availability may have also contributed to the ultimate uptake of HIV POCT. Finally, only one new reactive POCT was determined among participants. It is possible that our recruited population was already served by other testing and care services, or that HIV rates may be higher among those who did not agree to testing.

As we move towards scaling up HIV testing opportunities among at-risk groups, there is a need to develop novel models of community-based testing that draw on the strengths of peer involvement to reach individuals who may not seek testing and treatment in conventional health care settings. The PROUD study has shown high uptake of community-based and peer-administered HIV POCT among PWUD at highest risk of HIV acquisition. Future community and peer-based approaches should be implemented towards specific at-risk communities, including women and those involved in sex work, who may face challenges in accessing conventional clinic-based HIV-testing and treatment services.
